# *Quanty4RIXS*: a program for crystal field multiplet calculations of RIXS and RIXS–MCD spectra using *Quanty*

**DOI:** 10.1107/S1600577518004058

**Published:** 2018-05-01

**Authors:** Patric Zimmermann, Robert J. Green, Maurits W. Haverkort, Frank M. F. de Groot

**Affiliations:** a Debye Institute of Nanomaterial Science, Utrecht University, 3584 CA Utrecht, The Netherlands; b Stewart Blusson Quantum Matter Institute, University of British Columbia, Vancouver, British Columbia, Canada V6T 1Z4; c Department of Physics and Engineering Physics, University of Saskatchewan, Saskatoon, Saskatchewan, Canada S7N 5E2; d Institute for Theoretical Physics, Heidelberg University, 69120 Heidelberg, Germany

**Keywords:** *Quanty*, RIXS, RIXS–MCD, crystal field, CFT

## Abstract

Some initial instructions for the *Quanty4RIXS* program written in MATLAB^®^ are provided. The program assists in the calculation of 1*s* 2*p* RIXS and 1*s* 2*p* RIXS–MCD spectra using *Quanty*. Furthermore, 1*s* XAS and 2*p* 3*d* RIXS calculations in different symmetries can also be performed. It includes the Hartree–Fock values for the Slater integrals and spin–orbit interactions for several 3*d* transition metal ions that are required to create the .lua scripts containing all necessary parameters and quantum mechanical definitions for the calculations. The program can be used free of charge and is designed to allow for further adjustments of the scripts.

## Abstract 2

Documentation of the *Quanty4RIXS* program is presented with instructions for setup and usage.

## 1. Introduction   

This paper provides a first guidance and explanations on the usage of the *Quanty4RIXS* program for the calculation of resonant inelastic X-ray scattering (RIXS) and RIXS magnetic circular dichroism (RIXS–MCD) spectra. *Quanty4­RIXS* is related to the *CTM4XAS* program (Stavitski & de Groot, 2010 ▸) through the underlying crystal field model, though the computational approaches are fundamentally different.

*CTM4XAS* is primarily used to calculate one-dimensional X-ray absorption spectroscopy (XAS) or X-ray emission spectroscopy (XES) spectra based on the Cowan approach (Cowan, 1981 ▸; Thole & van der Laan, 1988 ▸). *Quanty4RIXS*, on the other hand, is specifically designed to calculate two-dimensional RIXS and RIXS–MCD maps taking advantage of the Green’s function approach used by *Quanty* which we explain in §3. For further details on the underlying physics and modelling we refer to the corresponding publications from Haverkort *et al.* (Haverkort *et al.*, 2012 ▸, 2014 ▸; Lu *et al.*, 2014 ▸; Haverkort, 2016 ▸).

*Quanty4RIXS* is a graphical user interface (GUI) simplifying the creation of the 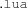 script which is used as an input file for *Quanty*. The 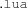 script is the standard input file for *Quanty* containing all parameters, quantum mechanical operators and definitions used for the calculation. (Examples of the different elements can be found at http://quanty.org/documentation/start.) The created 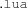 script can either be executed directly on a local machine using *Quanty4RIXS* to initiate the calculation, or the 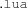 file can be saved and transferred to a high-performance computer to run the calculation.

*Quanty4RIXS* is written in MATLAB^®^ and the source code is published ‘as is’ to make it available to the scientific community. It can be altered and adjusted to meet the user’s own requirements as long as the original author (Patric Zimmermann, Utrecht University, 2017) is mentioned along subsequent versions.

## 2. Setup   

In order to run the program, MATLAB^®^ version 2016a or newer must be used. In older versions some GUI elements are not present, leading to errors. To enable the program to start the calculations locally, *Quanty* must be installed on a local machine. To obtain the latest version and further information on the usage of *Quanty* in the 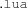 scripts, visit http://www.quanty.org.

**Figure 1:**
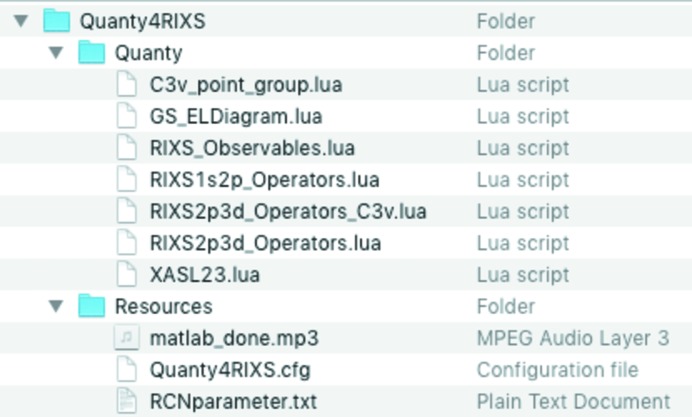
*Quanty4RIXS* repository folder.

*Quanty4RIXS* needs several files, which are stored in the repository directory as shown in Fig. 1 ▸. This repository folder is called 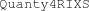 and its path, on a Mac, should be the user’s home directory 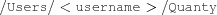 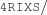 or, on Windows, 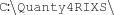. The configuration file 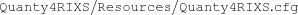 in the repository stores the paths to the *Quanty* binary and all settings, and therefore the folder’s permissions must be set to allow write access.

If the repository folder or the *Quanty* binary is not found in the default path the program will ask on first startup for the paths, which can then be selected accordingly.

## 3. The RIXS approach in *Quanty*   

In this section we describe the approach taken in the underlying program *Quanty* for the computation of RIXS spectra upon calling the 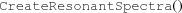 function. *Quanty* is an exact diagonalization program designed to take advantage of the sparse operators and wavefunctions present in many correlated electron problems.

Of relevance for *Quanty4RIXS*, *Quanty* calculates the RIXS intensity *I* for a given initial state, 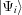 as 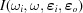 = 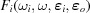, where 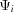 is the correlated many-body wavefunction, which is a linear combination over the various arrangements of electrons in the orbital, and 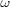 = 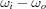 is the energy transfer between the incoming (*i*) and outgoing (*o*) photons with polarizations 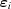 and 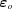, respectively.

To calculate the RIXS intensity for a given temperature *T* one computes the total intensity as a weighted sum over the contributing states as
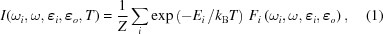
where 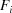 are the RIXS intensities for a given state 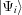 with energy 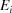, which is scaled according to the Boltzmann distribution using its energy 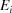, and *Z* is the partition function. In the following, however, we describe only the case for 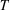 = 0 K.

With 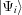 denoting a particular initial state of the system with energy 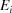 determined by the Hamiltonian *H*, one can describe the RIXS intensity by
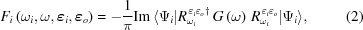
where
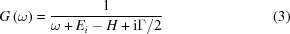
is the RIXS Green’s function with Γ as the final-state energy width due to the finite final-state lifetime. The effective RIXS operator 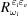 is given by (Haverkort, 2010 ▸)
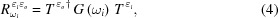
where
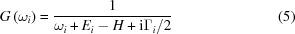
is the XAS Green’s function with 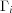 as the RIXS intermediate state energy width due to the finite core hole lifetime. Hence, the sandwiched expression in equation (2) translates to
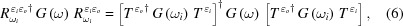
which can be explicitly rewritten as
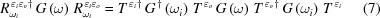
because 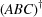 = 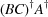 = 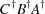.

This then describes the entire two-step RIXS process where a core hole is created by a transition operator 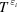 and subsequently annihilated locally by 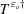 after the lifetime determined by the core hole width 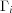. Depending on the type of RIXS studied, the absorption operator 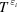 can describe, for example, a dipole 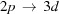 or quadrupole 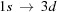 transition. Similarly the emission operator 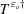 may induce a 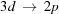 or a 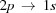 decay transition.

*Quanty* first calculates the initial state 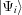 using a Lanczos-based iterative diagonalization. The state 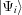 can be the ground state, or a low-energy state that is thermally populated (a block Lanczos algorithm is used to calculate a set of the lowest-energy eigenstates).

Next, *Quanty* calculates 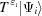 and uses this as a starting wavefunction to diagonalize *H* in a small Krylov basis [usually dimension 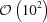]. Within this small Krylov basis, a quick inversion can be performed to obtain 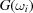.

This enables the wavefunction to be computed, 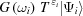, and transformed back to the original (large) basis.

Next, *Quanty* computes 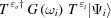 to replace 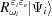 in equation (2) as a starting wavefunction to perform the final Lanczos-based diagonalization of *H* to obtain the RIXS spectral function for the state 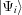.

For a finite temperature 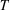 > 0 K or degenerate ground states, the process can be repeated for different initial states 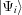 and the results weighted and summed as given in equation (1). For this purpose the 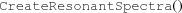 function does accept also a list of initial states 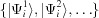 instead of just one initial state 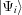 as starting wavefunction. This means that it is repeating the procedure described above, calling the function internally for each of the different states in the list. The result is then a combined RIXS map as shown, for example, in Fig. 8.

## 4. Using *Quanty4RIXS*   

The program can currently create *Quanty* input files for XAS, RIXS and RIXS–MCD calculations with circular polarized light. In other words, it creates a 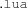 script with all the required parameters and definitions which are then used as input for *Quanty*. The job can be executed on a local machine or, for more time-consuming jobs, the script can be saved and then copied to any other computer that has *Quanty* installed.

**Figure 2:**
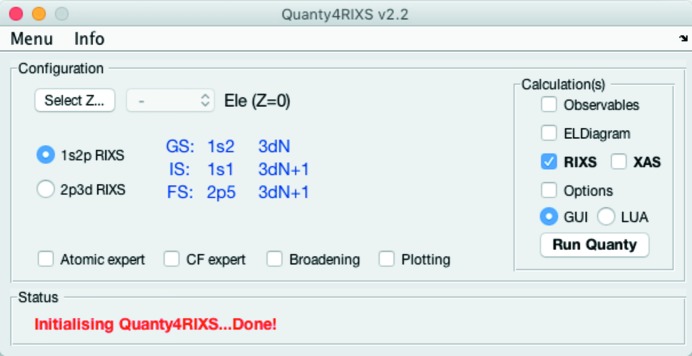
*Quanty4RIXS* main panel.

In the following we first give a short overview and some instructions on how the GUI can be used. The main panel of *Quanty4RIXS* as it looks after start-up is shown in Fig. 2 ▸. Here, one can select the ion configuration and the type of the calculation. When the 3*d* transition metal and its ionization level is selected the three corresponding RIXS electron configurations for the ground state (GS), the intermediate state (IS) and the final state (FS) are displayed.

Currently 1*s* XAS and 1*s* 2*p* RIXS for hard X-rays, and 2*p* 3*d* RIXS and 2*p* XAS calculations for soft X-rays are implemented. Additionally the expectation values (Observables) and an energy-level diagram (ELDiagram), also called the Tanabe–Sugano diagram, can be calculated. The Options tick box can be used to adjust the number of microstates to be computed, counted from the state lowest in energy.

**Figure 3:**
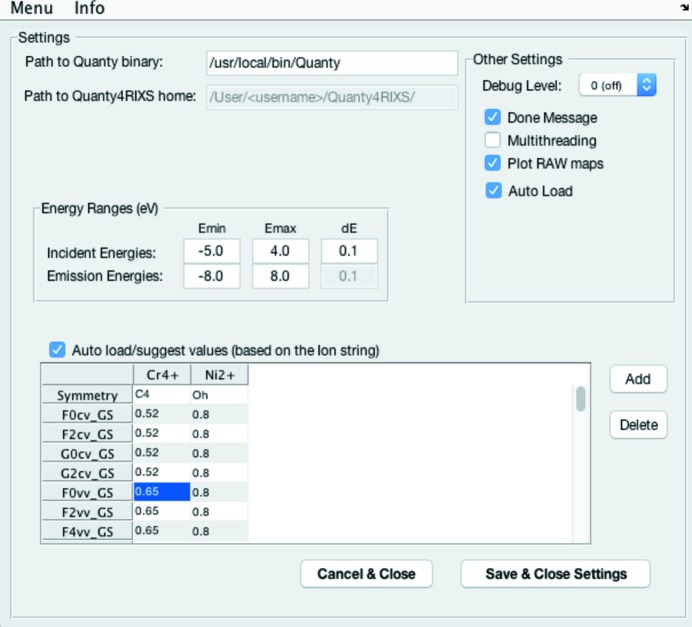
*Quanty4RIXS* settings menu.

The values for the energy ranges and the step width used in the calculation can be chosen and saved *via* the settings menu (Menu 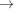 Settings) as shown in Fig. 3 ▸. Here it is advisable to start with a step width *dE* larger or equal to 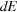 = 0.1 eV, because the finer the grid the more points of the spectrum have to be calculated. Hence, a finer grid will dramatically increase the calculation time and also the required memory. (We recommend 16 GB RAM or more.)

The Multithreading option can be used to split the calculation into two parts. If activated, a separate 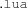 file is created for each circular polarization [left circular polarization (LCP) and right circular polarization (RCP)] which are then calculated in parallel. On multicore systems this significantly reduces the computation time. When disabled only a single 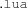 file is created. The option Auto Load refers to a procedure that automatically loads the result of a calculation into the plotting panel (*cf*. §4.6).

Apart from the relevant descriptions of the electron configuration, the Hamiltonians and crystal field parameters, the script’s header section contains all required atomic parameters (spin–orbit, Slater) for the selected 3*d* transition metal ion. The Settings panel also allows presets to be saved for the Slater and crystal field scaling parameters. The saved parameters are identified by the corresponding ion configuration.

The atomic, crystal field and broadening parameters are discussed in the following.

### 4.1. Atomic parameters   

The atomic parameters for several 3*d* transitions metal ions are stored in the 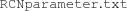 file. The values in the RCN file, for the Slater coefficients for exchange 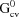, 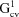, 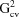, 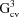, and Coulomb interaction 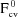, 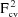, 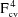, are the calculated Hartree–Fock (HF) values. The indices ‘cv’ are a placeholder for the corresponding shells, *e.g.* 1*s* 3*d* for the 1*s* 2*p* RIXS intermediate state. The contents of the RCN file have been calculated in advance with the Cowan program which is a part of *CTM4XAS* (Stavitski & de Groot, 2010 ▸) and as described by Cowan (Cowan, 1981 ▸) and Thole *et al.* (Thole & van der Laan, 1988 ▸). In principle, other ions can also be implemented; however, this requires adding the relevant information to the 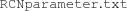 file and also some adjustments to the MATLAB^®^ code.

**Figure 4:**
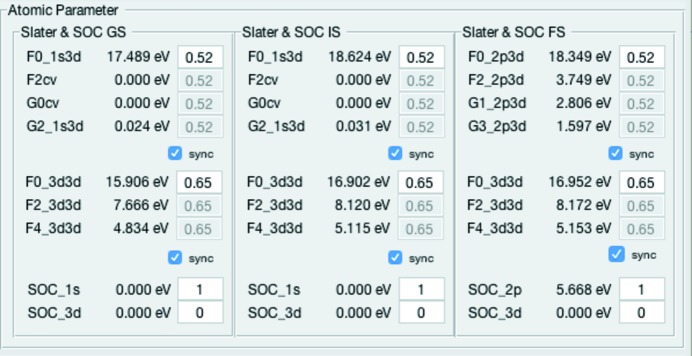
Atomic Parameter panel with values for the Slater coefficients (F_cv_ and G_cv_) and spin–orbit interaction.

The values can be scaled using the Atomic Parameter panel as shown in Fig. 4 ▸. Based on empirical knowledge they are usually reduced to 80% of the HF value to match the atomic values derived in experiments (Stavitski & de Groot, 2010 ▸) (*e.g.* 1.0 in the GUI means 100% of the HF value; 0.8 means 80% HF empirically equal to 100% atomic value; thus a scaling of 0.64 set in the GUI corresponds to 64% HF = 80% atomic). The spin–orbit interaction ζ is not screened and thus a scaling of 1.0 corresponds to 100% of the atomic value (Stavitski & de Groot, 2010 ▸). Due to the fact that all calculations only consider local contributions explicitly, the reduction of the Slater integrals can also be used to approximate hybridization effects (*e.g.* charge-transfer) as described by de Groot. (1991 ▸).

### 4.2. Crystal field parameter   

**Figure 5:**
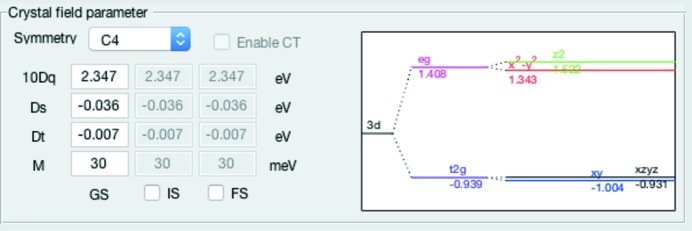
Crystal field parameter panel with symmetry and crystal field values.

The panel for the Crystal field parameter shown in Fig. 5 ▸ can be used to select a symmetry and set the corresponding crystal field parameters. The corresponding crystal field calculations are all performed with *Quanty*. Currently available symmetries are *O*_*h*_, *D*_4*h*_, *C*_4_ and *C*_3*v*_. To be precise, since *O*_*h*_ and *D*_4*h*_ are higher symmetry subsets of *C*_4_ symmetry, those three symmetries are all calculated in *C*_4_ and the reduction to higher symmetry is achieved by setting some parameters to zero. The crystal field parameter in *O*_*h*_ is 10*Dq*; in *D*_4*h*_ and *C*_4_ symmetry additionally *Ds* and *Dt* are non-zero. The parameter 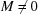 represents the magnetic exchange field (molecular magnetic field) implying *C*_4_ symmetry. (Note, the default is 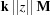.) If not modified the crystal field values are identical in the ground, intermediate and final state; ticking the box below enables them to be changed individually. Note that the explicit calculation of charge transfer is not yet implemented, but as mentioned above it can be approximated by a reduction of the Slater integrals.

The crystal field parameter panel also displays an approximated single-electron term scheme for the chosen crystal field parameters in *C*_4_ symmetry to illustrate the splittings of the 3*d* shell [the formulae can be found elsewhere (de Groot & Kotani, 2008 ▸)].

### 4.3. Broadening   

**Figure 6:**
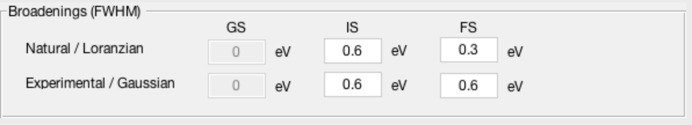
Broadening panel in *Quanty4RIXS*.

In the Broadening panel shown in Fig. 6 ▸ the user can choose the broadening values in eV, given as full width at half-maximum (FWHM). The ground state has an infinite lifetime, thus no natural broadening is applicable. The given broadening values are, together with the other parameters, written to the header section of the 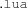 file. The experimental broadening in the current version is not used for the calculation; only the natural broadenings are applied to the spectra as parameters in the Green’s function approach (*cf*. §3). However, since all values are written to the script, when needed one can adjust the script to add an experimental broadening as a convolution between the natural spectrum and a Gaussian.

### 4.4. Creating a .lua script file   

The creation of the 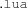 scripts for *Quanty* is based on the module-templates in the programs repository folder as shown in Fig. 1 ▸. The subdirectory *Quanty* contains several module-templates as 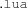 files, each containing a specific section to be used to assemble the final script. In principle these files can be altered as needed; however, some parts of the final script are dynamically created by the program, so one has to ensure a consistent naming of all used variables. The Resources folder contains the atomic parameter file 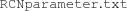, as discussed in §4.1, and the configuration file 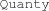 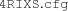 for the program. Hence the repository folder is crucial for the creation of the scripts.

With GUI mode selected in the main panel, clicking the Run Quanty button opens a file browser window where the path and filename for the output files must be given (spaces should be avoided in the path). Once the folder is chosen, the internal function 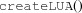 is called which assembles the final 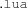 script. Some parts of the 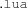 script are created dynamically based on the selections made in the GUI, but most parts of the script are assembled from the sections defined in the module-templates located in the repository folder. These module-templates can be altered when needed, though advanced changes are likely to also require some adjustments to the MATLAB^®^ code.

The resulting 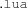 script is then saved in the selected folder together with a 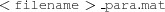 file, which holds a copy of the parameters as they are written to the 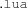 script. (Note: the 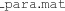 file is required for the plotting routines in the Plotting panel.)

The structure of the final 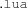 script is as follows:

(i) The header contains the ion information and the corresponding parameters, *e.g.* Slater, SOC, CF parameters, all for GS, IS, FS; and the path and filename used for the calculated maps (the latter is convenient when the 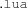 script is executed on an HPC computer).

(ii) Definitions of the XAS- and XES-transition operators, the Hamiltonians for the three states (GS, IS, FS) and polarizations; the Hamiltonians and the transition operators (TXAS, TXES) are hereby defining the entire two-step photon-in photon-out RIXS process.

(iii) Dynamic section performing the actual calculations of the spectra, ELDs or Observables. The *Quanty* function 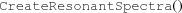 is used here to calculate the RIXS spectra. The Green’s function approach then yields the two-dimensional RIXS map implying that everything is a ground-state property.

Using the final confirmation window the 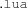 script is then used to start the calculation with *Quanty*. If cancelled, the files are just saved allowing the script to be transferred to another computer or changes to the script made.

### 4.5. Running a .lua script with *Quanty*   

There are two ways to start a calculation with *Quanty* using *Quanty4RIXS*. Clicking the Run Quanty button then triggers either of the following two methods:

(1) GUI mode selected in the main panel creates the script and starts the calculation.

(2) LUA mode selected asks for any prepared 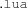 script to be executed with *Quanty*.

Method (1) was already mentioned in the previous section, where it directly starts the calculation based on the selections made in the GUI. Method (2) is meant as a simple click-and-run approach for any 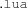 script that has been created with the GUI before or elsewhere. In this case the Run Quanty button opens a directory window where the user can select a prepared 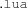 script for *Quanty*. This can be used to run a 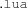 file that was altered manually or to run an arbitrary 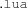 script for *Quanty* that was not created with the GUI. The selected file is then used as an input file for *Quanty*. (Note: this can cause the plotting procedure discussed in the following to no longer work.)

### 4.6. Plotting the results   

**Figure 7:**
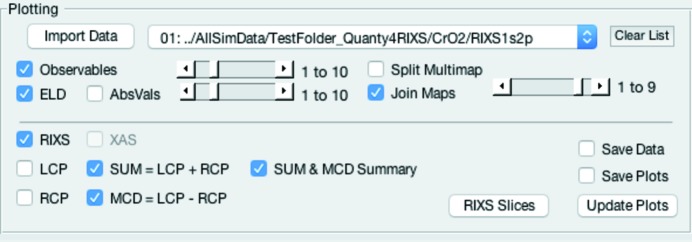
Plotting panel in *Quanty4RIXS*.

Here we introduce the Plotting panel, which is shown in Fig. 7 ▸. Owing to the complex file structure of the calculated RIXS maps when more than one microstate is involved, one has to split the resulting data for each microstate and sum them accordingly. This enables, for example, all microstates to be quickly reviewed and compared at once, and a normalized linear combination to be realized before considering a Boltzmann distribution to model a temperature dependency. To achieve this one can calculate and use the expectation values (Observables) to determine the scaling factors for each microstate [see equation (1)].

**Figure 8:**
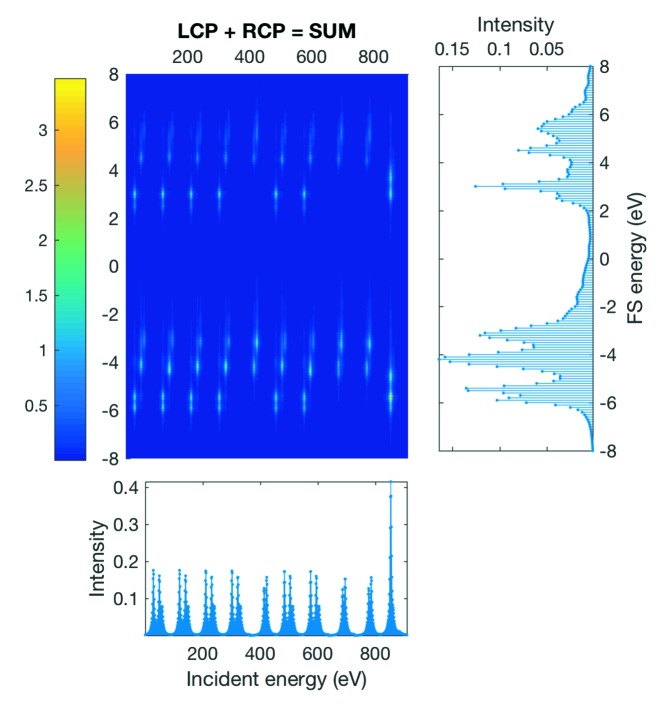
Example 1*s* 2*p* RIXS calculation with ten microstates.

Fig. 8 ▸ shows a raw RIXS map with ten microstates as it is calculated by *Quanty*. One can see that in the raw data each individual RIXS map, for each microstate, is merged into one large map showing all microstates at once (we call them here ‘Multimaps’).

The XAS projection onto the incident energy axis (bottom panel in Fig. 8 ▸) directly reveals that not all microstates yield the same RIXS map. (Note: the values on the incident energy axis are for a Multimap, just indices and not energy.)

The plotting panel enables the user to quickly review the result of the calculation and each individual microstate without needing to write a complex plotting routine. For that purpose sliders were added. They can be used to choose how many microstates are used to join the maps (Join Maps). This will equally add all RIXS maps up to the given microstate. With Split Multimap selected, it can also be used to split the raw data and plot only one specific microstate. In spite of the various plots possible, all plots are rather rudimentary, because its main purpose it to quickly plot the calculated RIXS and RIXS–MCD maps for review.

**Figure 9:**
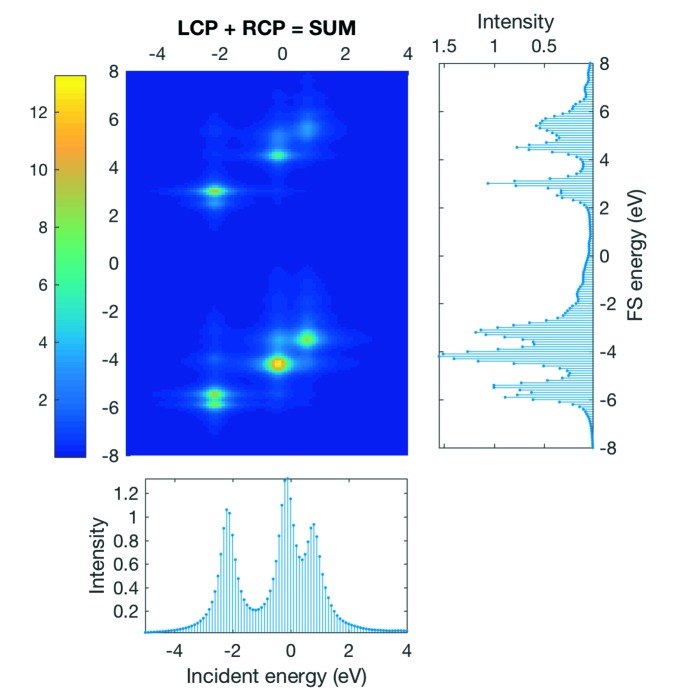
Arbitrary example for a 1*s* 2*p* RIXS map showing the *K* pre-edge for a Cr^4+^ ion.

An example of one individual 1*s* 2*p* RIXS map using the join function is shown in Fig. 9 ▸. The RIXS map shown Fig. 9 ▸ is the total RIXS as a sum of the the LCP and RCP, where each is the sum 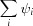 over the lowest nine microstates 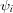 (with 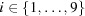) and therefore represents a possible variant of the 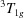 ground-state symmetry (3 × 3 = 9) of a Cr^4+^ ion.

**Figure 10:**
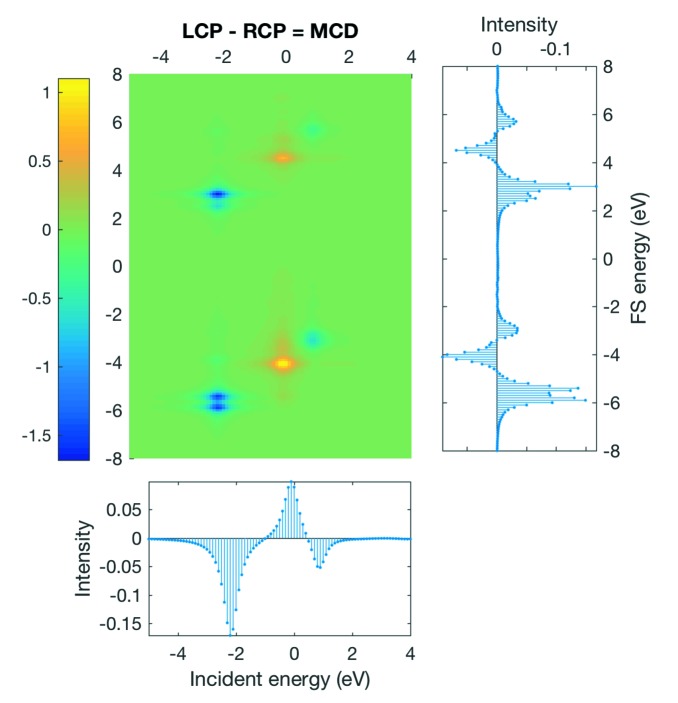
Example of a 1*s* 2*p* RIXS–MCD map of the *K* pre-edge for a Cr^4+^ ion.

Furthermore, the magnetic circular dichroism (MCD) can also be calculated. Owing to symmetry considerations the calculations are performed in spherical coordinates for which the LCP and RCP can each be directly represented by a single quadrupole operator, each corresponding formally to one spherical harmonic operator 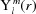,
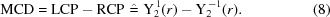
An arbitrary example of such a 1*s* 2*p* RIXS–MCD map together with the projections onto its axes is shown in Fig. 10 ▸. The RIXS–MCD map shown corresponds in this example to only the first microstate 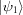. Subsequently, for an accurate modelling, a linear combination of all involved microstates is required. For MCD calculations under ambient conditions (*T* > 0 K) it is appropriate to use the Boltzmann distribution to produce a reasonable result [equation (1)].

**Figure 11:**
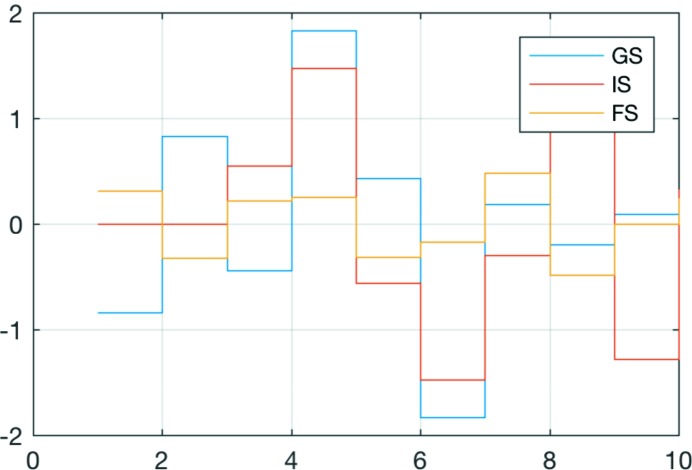
Example plot of the expectation values (Observables) showing here the values of 〈*S*_*z*_〉 for the lowest ten microstates for the three RIXS steps (GS, IS, FS).

Here one can use the Observables option to compute the expectation values of the Hamiltonian 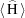 to obtain the relative energy of each microstate, determining the Boltzmann coefficients. Using the Font Observables option one can calculate the quantum mechanical expectation values of any defined operator 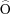 for each micro state 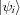:
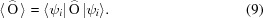
Another example is the expectation values 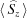 for which Fig. 11 ▸ shows a plot for the lowest ten microstates for the three RIXS steps (the ground, intermediate and final state; GS, IS, FS). Several observables are already implemented to be directly computed. They include, for example, 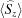, 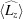, 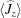, 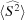, 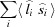 and others. In principle, any operator that is defined in the script can be used to calculate its expectation value. When required, additional operators can be defined for this purpose.

## 5. Concluding remarks   

We hope that the above descriptions are helpful for those who want to use the software. We want to explicitly point out that adding more or changing the existing 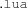 modules in the repository is possible as long as the variable names are used consistently. Simple changes can be implemented without needing to adjust the MATLAB code; however, more advanced changes such as, for example, adding other symmetries will most likely require changes to the *Quanty4RIXS* routines as well.

Particularly for RIXS–MCD spectra the results can dramatically change even for very small parameter changes. To get started we recommend to begin with the atomic case. From there one has to evaluate each parameter individually to approach the specific system under study. Here, additional information, for example from density functional theory calculations, can be advantageous to estimate good values.

The complete MATLAB source code for *Quanty4RIXS*, the repository folder and a folder with all dependency graphs (as PDF) illustrating the connection of all functions has been published at https://git.science.uu.nl/P.Zimmermann/Quanty4RIXS/. For full access, a guest account can be created (*via* contacting the authors).

The code has been thoroughly tested on Apple OS X 10.11 and in part also on Windows 7. In principle it should work on any operating system as long as the setup requirements described in §2 are met. We used and recommend OS X because it has in general shown a better performance and less memory consumption.

All sources are made available ‘as is’ and it can be altered to the user’s liking as long as a reference to the original author (Patric Zimmermann, Utrecht University, 2017) is given. For subsequent scientific work we appreciate citation of this paper.
